# Ganglion cell layer segmentation and the two-flash multifocal electroretinogram improve structure function analysis in early glaucoma

**DOI:** 10.1007/s00417-017-3722-x

**Published:** 2017-08-04

**Authors:** Livia M. Brandao, Anna A. Ledolter, Matthias Monhart, Andreas Schötzau, Anja M. Palmowski-Wolfe

**Affiliations:** 1 Department of Ophthalmology, University of Basel, Basel, Switzerland; 2 Universitätsspital Basel Augenklinik, Mittlere Strasse 91, 4031 Basel, Switzerland; 3 Department of Ophthalmology, Medical University of Vienna, Vienna, Austria; 4 Carl Zeiss Meditec, Feldbach, Switzerland

**Keywords:** Multifocal electroretinogram, Standard automated perimetry, Optical coherence tomography, Ganglion cell, Glaucoma

## Abstract

**Purpose:**

To improve structure-function analysis in primary open-angle glaucoma (POAG) by including the two-global flash multifocal electroretinogram (2F–mfERG) and macular ganglion cell layer segmentation.

**Methods:**

Twenty-five glaucoma patients (six pre-perimetric (PPG), 19 POAG) and 16 controls underwent 2F–mfERG, optical coherence tomography (OCT), and standard automated perimetry (SAP). For 2F–mfERG, the root mean square was calculated for the focal flash response at 15–45 ms (DC) and the global flash responses at 45–75 ms (IC1) and 75–105 ms (IC2). For OCT, macular total thickness (mT) and ganglion cell-inner plexiform layer (GCIPL) thickness were analysed. Values from the central 10° and 15° of 2F-mfERG were compared to the corresponding areas from OCT and visual field.

**Results:**

Both PPG and POAG had significantly lower mfERG responses in the central 10° and 15° than the control group. Of the glaucoma patients, 30.7% (three PPG, five POAG) showed central mfERG and GCIPL reduction without a SAP defect in the central 15 degrees. Four patients had a central SAP defect associated with a reduced GCIPL without any detectable dysfunction on mfERG. MfERG DC and IC2 were larger with increased mT (*p* ≤ 0.02), but GCIPL only related positively to IC2 (*p* = 0.027). SAP sensitivity also increased with thicker mT but not with GCIPL (*p* < 0.03 and *p* = 0.35). DC, IC2, and GCIPL could best differentiate glaucoma from control (AUC values: 0.897, 0.903, and 0.905).

**Conclusions:**

Structure function analysis in glaucoma can be improved when the GCIPL thickness as well as the 2F–mfERG is included as these measures complement information obtained by SAP.

## Introduction

Blindness due to glaucoma is still common all around the world and incidence has continued to rise [1]. Because the disease can be asymptomatic for a long time, early diagnosis is still limited as it had not been possible to ascertain which abnormality (structural or functional) presents first in the natural course of the disease [2].

Ganglion cells are the first to be affected in glaucoma, and approximately 50% of them are concentrated in the parafoveal area [3]. Optical coherence tomography (OCT) has been largely used to evaluate structural integrity in glaucoma patients. Evaluating total macular thickness appears to be less sensitive than the ganglion cell-inner plexiform layer thickness (GCIPL) in POAG [4, 5]. Analysis of GCIPL thickness has a similar performance to correctly diagnose glaucoma when compared to the retinal nerve fiber layer (RNFL) [6, 7]. A limitation of structural OCT analysis is that, according to a model proposed by Hood et al. [8], the OCT may still be normal in the presence of cell loss, if the individual’s number of cells at onset was on the high end of normal.

Standard automated perimetry (SAP) has been applied to the diagnosis of POAG for decades, although SAP only shows dysfunction once a significant number of cells have already been lost [3]. Thus, structural changes in the ganglion cell layer can precede functional changes in SAP [3, 9]. Hood and Kardon proposed a structure function model in which ganglion cell loss and SAP sensitivity have a linear relationship [10].

Animal models have shown that elevated IOP alters inner retinal function prior to the occurrence of structural damage [11]. In an attempt to discover glaucomatous dysfunction at an earlier stage, the multifocal electroretinogram (mfERG) technique has been explored in POAG [12–18]. The mfERG introduced by Sutter and Tran [19] allows for simultaneous functional testing of multiple retinal locations and provides a high resolution topographic examination of retinal function. The contributions of the inner retina to mfERG have been studied [12, 20–25]. The first order response of mfERG originates predominantly (but not exclusively) from the outer retina, whereas the higher order components are primarily (but not exclusively) attributed to inner retinal function. Analysis of the “ONH component” [20], the photopic negative response [17, 26, 27], the oscillatory potentials [28], global flash paradigms [12, 21, 22, 28–31], and stimulus contrast adaptations [22, 24, 32, 33] attempted to increase the inner retinal contributions to the mfERG response, and thus its sensitivity to glaucoma. In stimuli with global flashes, responses evoked by the influence of a previous stimulus on that to a global flash is thought to reflect a contribution from the inner retina [28, 30, 34]. In the global flash stimulus, the direct component (DC), that is the response to the M-Sequence step, is also enhanced through adaptive effects of the global flashes on the response to the m-sequence step.

Our group has shown that the two-flash multifocal ERG (2F–mfERG) demonstrated a good sensitivity in identifying glaucomatous retinal dysfunction [12, 14], particularly in the central 10° for the first (IC1) and second (IC2) induced components [15, 35]. Central full macular thickness (mT) was also associated with 2F–mfERG response in this area [16].

In this study, we assess to what extent including the GCIPL analysis, in addition to mT measures, as well as the two-flash multifocal electroretinogram, together with SAP, improves structure-function analysis in glaucoma. To the best of our knowledge, this is the first study in humans to combine the 2F–mfERG and macula layer segmentation in glaucoma patients.

## Materials and methods

The study protocol was approved by the ethics committee of the University of Basel, and informed consent was obtained from all participants before the examination. All procedures followed the tenets of the Declaration of Helsinki.

All individuals recruited to this study presented a visual acuity of 0.8 or better and a refractive error between ±6 diopters of hyperopia or myopia.

POAG patients were recruited directly from the glaucoma specialist consultations at the university eye clinic and had a glaucomatous optic neuropathy on fundus examination, as well as a controlled IOP under use of topical medication. Presence of localized thinning of the neuroretinal rim was confirmed on OCT with at least one red sector or two yellow sectors on the 12 sector clock hour RNFL thickness map (less than 1 and 5% of the normal population, respectively). POAG patients had a visual field defect reproducible in at least three tests (Octopus 101, G2 protocol), which corresponded to the RNFL OCT defect.

Preperimetric glaucoma (PPG) eyes presented with optic nerve glaucoma abnormalities and the same OCT defect criteria described above, but a normal visual field [mean defect (MD) under 2.2 and square root of loss variance (sLV) under 2.5].

Exclusion criteria: systemic diseases and/or regular use of medications that could influence the eye (i.e. antidepressant, anticonvulsive), as well as previous ocular surgeries (including cataract surgery).

Each participant was given a complete ophthalmic evaluation including corrected visual acuity, Goldmann tonometry, slit lamp and fundus examination. Subjects underwent SAP, 2F–mfERG, and OCT testing as described below. In all controls and 19 eyes these tests were obtained on the same day. If for any reason these tests could not be performed in the same day (*n* = 7), interval between tests did not exceed 6 months (median 2 months). Analyzed data corresponded to the central 10 and 15 degrees.

**Fig. 1:**
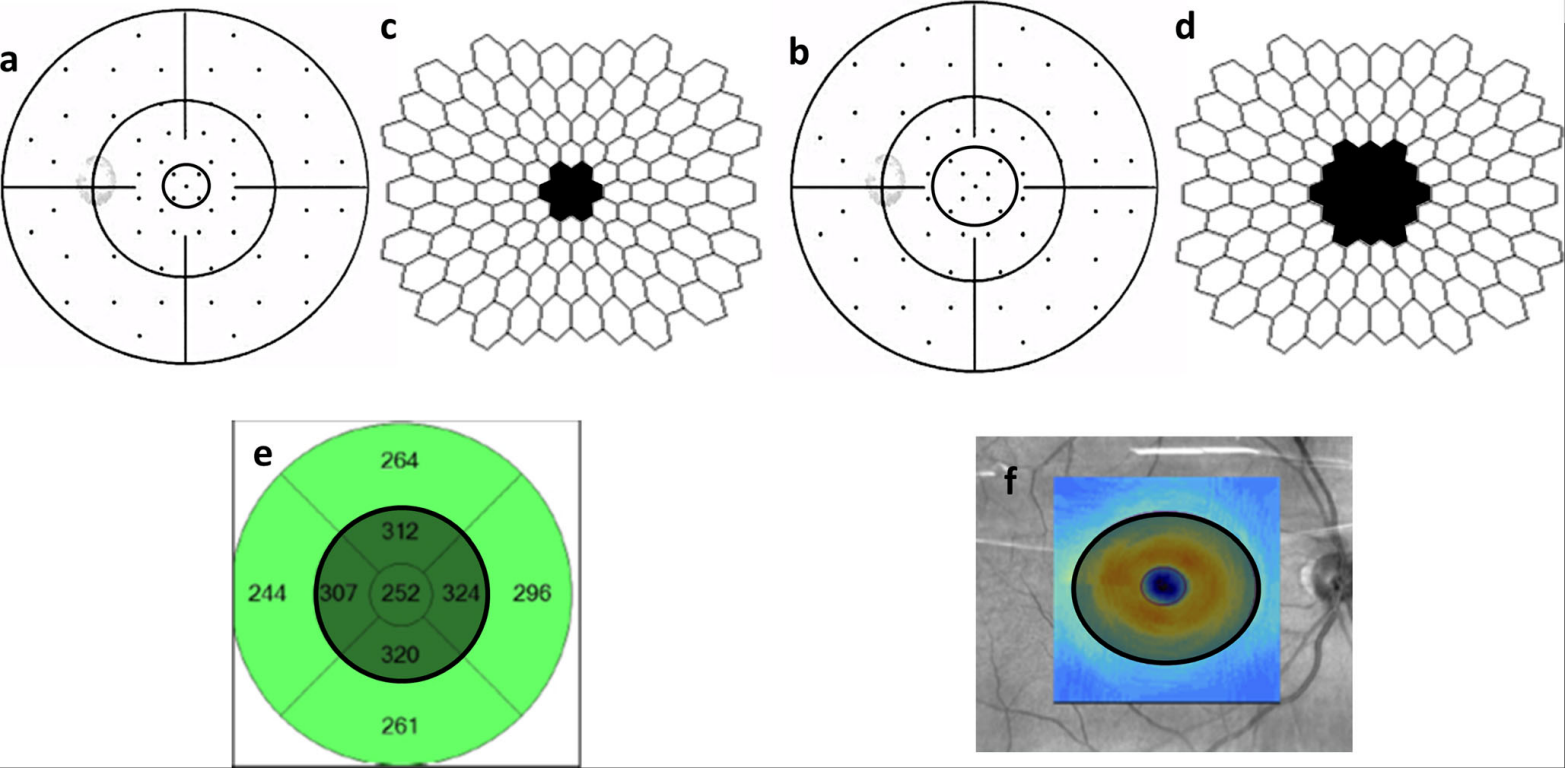
This figure gives an overview of the different examinations compared for the individual analysis shown in the circled areas in a right eye. Corresponding areas from the central degrees were analyzed for the central 10° to the left: SAP (a), 2F–mfERG (c), mT (e) and for the central 15° to the right: SAP (b), 2F–mfERG (d), mT (f)

SAP was performed using an Octopus perimeter (Octopus 101, G2 Program, Haag-Streit AG, Switzerland). Mean sensitivity (MS) and mean defect (MD) values were calculated for each stimulus location in dB and converted individually into linear units. For the central 10°, these focal linear values were averaged to form MS10° and MD10° for comparison to total mT as shown in Fig. 1 (left, a). Taking into consideration the slightly larger area included in the GCIPL measurement, an additional four focal linear values were included to form MS15° and MD15° (Fig. 1, right, b). This also takes into consideration the displacement of the retinal ganglion cells as described by Drasdo [36] and adapted for the G2 SAP according to calculations by Hood and Raza based on the Humphrey 10–2 SAP [37].

Figure 1 is a graphical depiction of the visual field points included in the respective analysis, a: for comparison to mT and 2F-mfERG and b: for comparison to GCIPL.

The 2F–mfERG was recorded using VERIS Science 6.2.2™, FMSIII (Electro-Diagnostic Imaging, San Mateo, CA, USA) with a Burian-Allen bipolar contact lens (Hansen Ophthalmics, Iowa City, IA, USA), through pharmacologically dilated pupils (Tropicamide 0.5%, Phenylephrine 1%, Spital-Pharmazie USB, Switzerland). The central 50° of the retina was stimulated by 103 hexagons, scaled with eccentricity. These flickered according to an m-sequence of 2^13^−1^ (L_max_ 100 cd/m^2^, L_min_ < 1 cd/m^2^). Each m-sequence step was followed by two global flashes (200 cd/m^2^) at an interval of 26 ms. A band-pass filter was set at 1–300 Hz. The total recording time of 10 min and 55 s was divided into 16 segments. During recording the summed response was continuously monitored and segments contaminated by ocular movements or poor signals were excluded and re-recorded. The artifact rejection technique was applied twice [38], and each focal response was filtered by segment (as suggested by the manufacturer) at 1–200-Hz. The root mean square (RMS) was calculated for the direct component at 15–45 ms (DC), and the two response components induced by the effects of the preceding focal flash on the response to the global flashes at 45–75 ms (IC1) and at 75–105 ms (IC2).

**Fig. 2:**
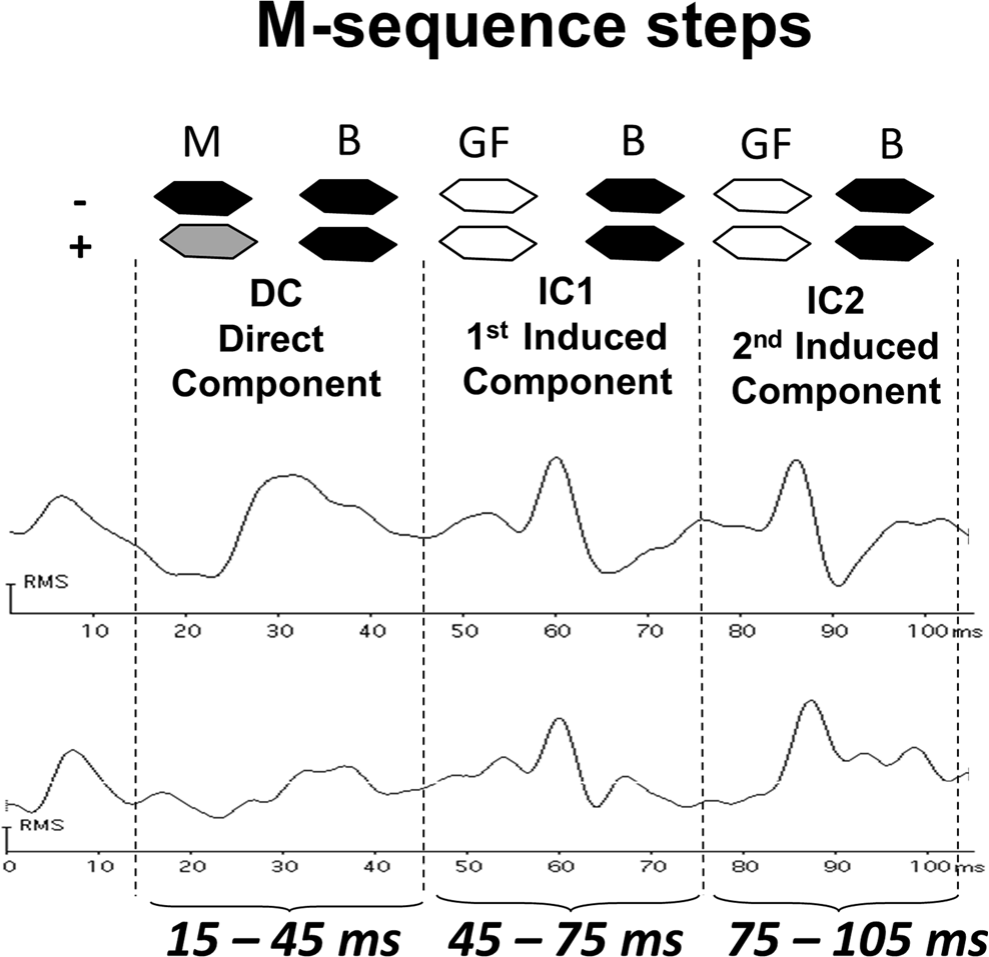
Schematic representation of the two-global flash mfERG stimulus and respective original wave forms from a control and a patient from the study. Legend: M: m-sequence step; B: black frame; GF: global-flash frame; DC: direct component; IC1: first induced component; IC2: second induced component

Figure 2 shows the two-flash stimulation protocol and original wave forms from one control and one POAG, with the components analyzed (DC, IC1 and IC2). RMS values from the central 7 hexagons (10°) were averaged, and compared to mT, MS10° and MD10° (Fig. 1, c). To correspond to the OCT area of GCIPL thickness (central 13.2° × 15.8°) a second mfERG RMS average was formed, including the 19 central hexagons (15°). This was then also compared to MS15° and MD15° (Fig. 1, d).

OCT images were obtained using the fast macular cube 512 × 128 protocol (Cirrus SD-OCT, Carl Zeiss, USA). Values for total macular thickness (mT) and GCIPL were calculated in microns as per Cirrus software (version 6.5.0.722; Fig. 1, e). The mT values used in this study corresponded to the 1- plus 3-mm diameter circles of the Early Treatment Diabetic Retinopathy Study (ETDRS) grid analysis from the Cirrus software, and correspond to the central ∼10°. The GCIPL thickness analysis of the Cirrus SD-OCT, encompasses the area of highest ganglion cell density [39], and consisted of an elliptical annulus (4.0 mm vertical and 4.8 mm horizontal diameter), corresponding to the central 13.2° and 15.8° (Fig. 1, f). The central elliptical area of the foveola is excluded (1 mm, corresponding to 3.5°), taking into consideration the displacement of the retinal ganglion cells which is largest at 1 mm where the displacement is about 0.62 mm [36]. At 4 mm eccentricity, this displacement becomes negligible (about 0.12 mm) [36] .

### Statistical analysis

One-way ANOVA (including Bonferroni as a post hoc test), was used to access differences between groups. To investigate the relationship between structure and function, linear mixed-effects models were performed. Function parameters (SAP sensitivity or mfERG response) were considered as the dependent variables while OCT measurements were the independent variables. Results were adjusted for age and gender (fixed effects) and expressed as regression coefficients (slope coefficient) with corresponding 95% intervals and *p* values. The coefficient was interpreted as the rate of change of the target variable, increasing the predictor by one unit. A *p* value <0.05 was considered significant, while a value <0.1 was considered a trend. Statistical analyses were performed using the statistical package R [40] (version 3.0.2) and SPSS (IBM statistics version 22). False discovery rate (FDR) adjustment was used to adjust *p* values for multiple comparisons. Specificity and sensitivity were assessed with a ROC analysis. All ROC curves were calculated using a binary classification of disease/no disease based on logistic regression including all proposed classifiers analyzed together: DC, IC1, and IC2 (for the central 10 and 15 degrees), GCIPL, total macula thickness, MS and MD (for the central 10 and 15 degrees). AUC and SE (AUC) calculations were done using SPSS version 22, taking into consideration age and gender, included as predictors in the regression model [41]. Thus, for each ROC analysis, first a logistic regression model was done in SPSS with the following predictors: “age + gender + variable.of.interest”. Then, based on the predicted values (in SPSS) of the logistic regression, ROC curve and AUC were calculated. These are based on nonparametric assumptions (Wilcoxon-statistics).

## Results

A total of 42 eyes were included: six PPG, 20 POAG, and 16 controls. Demographic details are presented in Table 1. Mean age was 61.1 years (SD ± 13.06) for the glaucoma group and 49.25 years (SD ± 7.02) for the controls. Age differed between POAG and controls (*p* < 0.01), but not between the PPG and controls (*p* = 0.068). Mean MD in POAG was 4.7 reflecting the early disease stage. Overall MD was significantly different between the glaucoma groups, reflecting the normal visual fields in PPG (*p* = 0.013). Mean MD also differed between POAG group and controls (*p* = 0.001).

**Table 1:** Demographic and Clinical Data. SD: standard deviation, CI: confidence interval; BVCA: best corrected visual acuity (decimal), IOP: intraocular pressure under topical medication (mmHg), CDR: cup-to-disc ratio, MD: mean defect (dB), PPG: preperimetric glaucoma, POAG: primary open-angle glaucoma. One-way ANOVA

Group		PPG (*n* = 6)	POAG (*n* = 20)	Controls (*n* = 16)	*p* value
Age (years)	Mean ±SD	63 ±15.9	60.6 ±12.4	49.2 ±7.02	*p* < 0.01
Gender	(M/F)	5/1	17/4	2/14	
BVCA	Mean (95% C.I.)	0.9 (0.8/1.1)	0.9 (0.9/0.9)	1.1 (1.0/1.0)	*p* < 0.001
MD	Mean (95% C.I.)	−0.08 (−0.6/0.4)	4.7 (2.7/6.7)	0.12 (−0.8/1.0)	*p* = 0.001
IOP	Mean (95% C.I.)	13.6 (12.0/15.2)	12.2 (11.0/13.3)	13.3 (11.8/14.7)	*p* = 0.435
CD	Mean (95% C.I.)	0.8 (0.7/.09)	0.74 (0.7/.08)	0.29 (0.2/0.3)	*p* < 0.001

## Analysis of the central areas: glaucoma x controls

### Sap

Neither MS10° (*p* = 0.096) nor MS15° (*p* = 0.083) differed between groups, even though the average linear sensitivity was smaller in POAG than in controls and PPG.

The same held true for MD, where the average defect was higher in POAG when compared to controls and PPG, but this did not reach a significance level (MD10°: *p* = 0.070 and MD15°:* p* > 0.07).

### 2F–mfERG

In contrast in the 2F–mfERG differed more between the groups

**Fig. 3:**
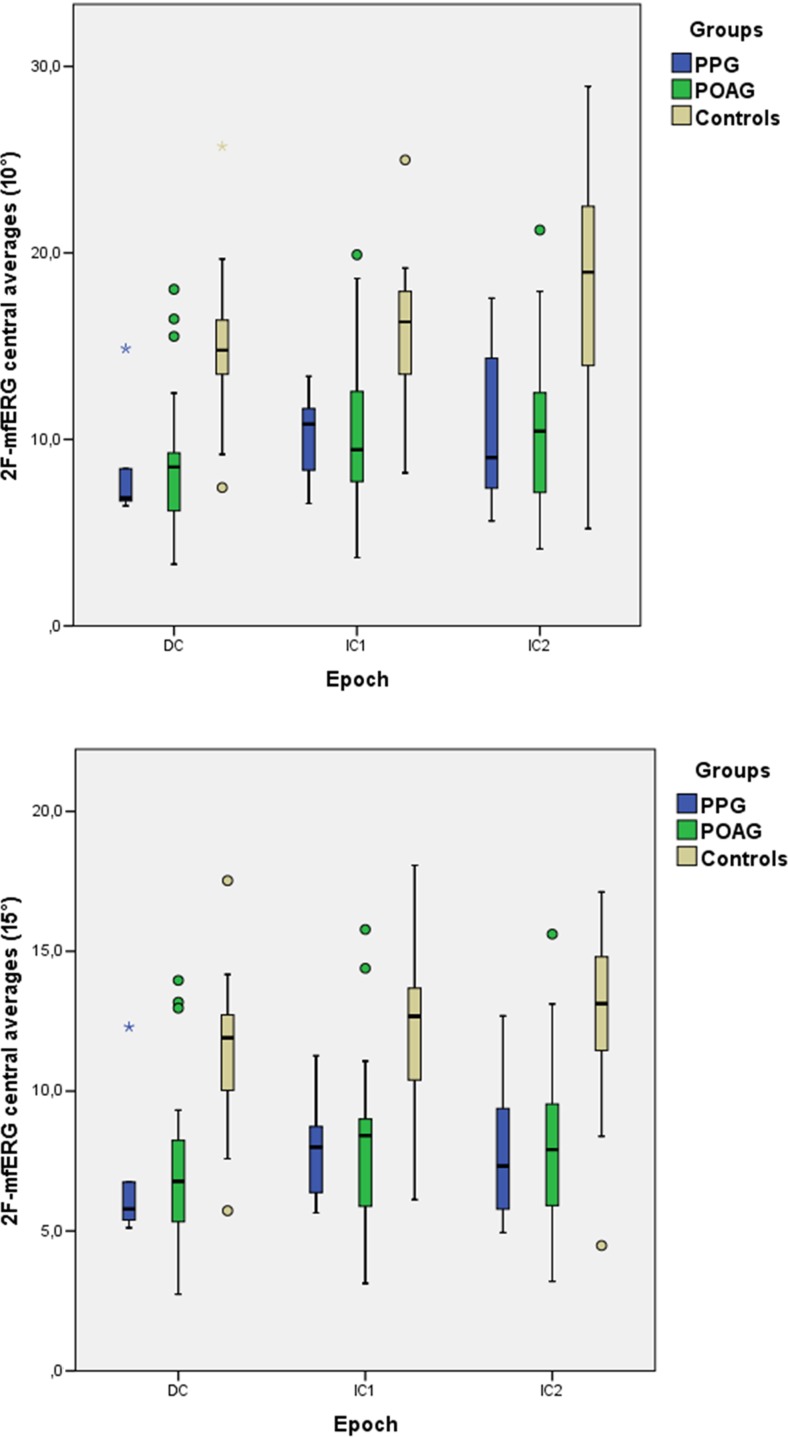
Boxplot distribution of the 2F–mfERG RMS response averages from the central 10° (top) and 15° (bottom) for PPG, POAG, and controls. Boxes represent the first quartile (lower end of box), median (band inside the box), and third quartile (upper end of the box). Ends of the whiskers represent the lowest data point (lower whisker) still within 1.5 interquartile ranges (IQR) of the lower quartile and the highest data point (upper whisker) still within 1.5 IQR of the upper quartile. For each RMS component response average (DC, IC1, and IC2): POAG group (left box), PPG group (middle box), and controls (right box). DC: direct component; IC1: first induced component; and IC2: second induced component. Symbols outside whiskers are considered outliers

Figure 3 summarizes the central 2F–mfERG results: when compared to control, MS10° from all epochs (DC, IC1, and IC2) were significantly decreased in POAG (all *p* ≤ 0.001) as well as in PPG (*p* ≤ 0.03). Neither DC, IC1, or IC2 differed significantly between POAG and PPG. RMS15° from all epochs were significantly decreased in POAG as well as PPG (*p* ≤ 0.022), but did not differ significantly between POAG and PPG (*p* = 1).

### Oct

Table 2 summarizes the OCT measurements: While mT did not differ significantly between the glaucoma groups or between glaucoma and controls (*p* > 0.10), GCIPL differed significantly between controls and both POAG: *p* < 0.001 and PPG: *p* = 0.012.

**Table 2:** Mean thickness values among groups and *p* values. SD: standard deviation, mT: full macular thickness, GCIPL: ganglion cell-inner plexiform layer thickness. The *p* values from one-way ANOVA

Group		PPG (*n* = 6)	POAG (*n* = 20)	Controls (*n* = 16)	*p* value
mT	Mean ± SD	302.6 ± 15.8	296.5 ± 18.7	308.4 ± 12.7	*p* = 0.105
GCIPL	Mean ± SD	68.2 ±102.6	65.4 ± 9.3	80.7 ± 4.7	*p* < 0.001

## Analysis of the central areas: structure x function

A linear mixed-effects model was used to assess the structure function relationship between mfERG and SAP or OCT. Association between SAP sensitivity and OCT was assessed using a linear model. Visual field values were converted to linear scale before averaging parameters. Table 3 summarizes these relationships that are described below:

**Table 3:** Structure function relationship expressed as regression coefficients and corresponding *p*-values (considering eyes from patients and controls pooled together). Regression coefficient is interpreted as the rate of change of the target variable, increasing the predictor by one unit. GCIPL: ganglion cell-inner plexiform layer, MS10° and MS15°: values from 10 and 15 degrees, respectively. All calculations were adjusted to age and gender. *: results from simple linear regressions. *p* values adjusted for multiple comparison with FDR

Test	Macular Thickness		GCIPL		MS 10°		MS 15°	
	Regression coefficient	*p* value	Regression coefficient	*p* value	Regression coefficient	*p* value	Regression coefficient	*p* value
2F–mfERG	0.05	<0.01	0.28	0.070	−0.002	<0.01	−0.002	<0.01
Epoch of mfERG								
DC	0.05	<0.02	0.07	0.231	−0.002	0.045	−0.002	0.045
IC1	0.03	0.095	0.05	0.327	−0.002	<0.01	−0.002	<0.01
IC2	0.05	<0.01	0.09	0.072	−0.002	<0.01	−0.002	<0.01
Macular Thickness	n/a	n/a	n/a	n/a	−0.30*	0.315	n/a	n/a
GCIPL	n/a	n/a	n/a	n/a	n/a	n/a	5.493*	0.391

### mfERG versus OCT

mT correlated significantly to RMS10° DC (*p* = 0.02) and IC2 (*p* = 0.009). For IC1, however, this positive correlation was just a trend (*p* = 0.09). This means RMS10° responses were larger the thicker mT.

GCIPL showed a significant positive correlation to RMS15° only in correlating IC2 to PPG (*p* = 0.027, control: *p* = 0.30; POAG: *p* = 0.52). DC had a positive trend only in the PPG group (*p* = 0.052).

### SAP versus OCT

MS10° was positively associated to mT for PPG (*p* = 0.029) but negatively associated in controls (*p* = 0.001). MD10° showed a negative association with mT in POAG (*p* < 0.001), a negative trend in PPG (0.078) but none with controls (*p* = 0.69).

There was no significant association between MS15° and GCIPL (*p* = 0.348), although MD15° showed a negative association (*p* < 0.001) only in POAG.

### mfERG versus SAP

MS10° showed a significant positive association with the 2F–mfERG RMS10° in PPG, POAG and controls for all epochs (DC, IC1, and IC2, *p* < 0.001). There was no significant association between MD10° and RMS10°.

Overall, MS15° was negatively associated with RMS15° in all epochs (*p* ≤ 0.017). In PPG DC showed a trend, while IC1 and IC2 were significantly associated at a level of *p* < 0.02). In controls we saw a trend (IC1 and IC2 *p* = 0.05 and 0.07, respectively) while this did not reach trend level for POAG.

We did not observe any significant relationship between MD15° and RMS 15°.

### Individual analysis of the central 15°

An individual analysis of the patients was performed for each eye tested (Fig. 1). As the GCIPL was the most sensitive OCT parameter, for SAP and mfERG we analyzed the central 15° for better comparison to the GCIPL. This encompasses the central 10°. Information on mT refers to the central 10° as stated in the methods section.

An abnormality in central area (15°) was defined as:

1. SAP: Presence of at least one point with a probability smaller than 0.5% in the MD deviation plot.
2. 2F–mfERG: RMS average outside the 95% quantile of controls in DC, IC1, and/or IC2.
3. OCT: Presence of at least one yellow or one red sector on the thickness map (≤5% and ≤1% of normal population, respectively) for mT and GCIPL, respectively.

The individual analysis showed that in one of 26 eyes, an eye from a patient with PPG, had no alterations in the macular OCT (neither mT nor GCIPL), the central mfERG, or SAP. Another PPG patient had alterations only in the mfERG. Of the remaining 24, 17 had both an abnormal mT and GCIPL; six patients had a normal mT measurement associated with a thinning of the ganglion cell layer (one PPG, five POAG) and one patient had a pathologic mT with a normal GCIPL (POAG). Among the 23 patients with GCIPL thinning, eight (34.7%) demonstrated pathologic central 2F–mfERG results associated with a normal central visual field, whereas four patients had a central visual field defect and a normal central 2F–mfERG. Three patients had alterations in both SAP and 2F–mfERG associated with a GCIPL thinning. Of all 26 patients, 30.7% (three PPG, five POAG) had central mfERG and GCIPL alterations, but no SAP defect in the central 15 degrees.

### mfERG x SAP x OCT

**Fig. 4:**
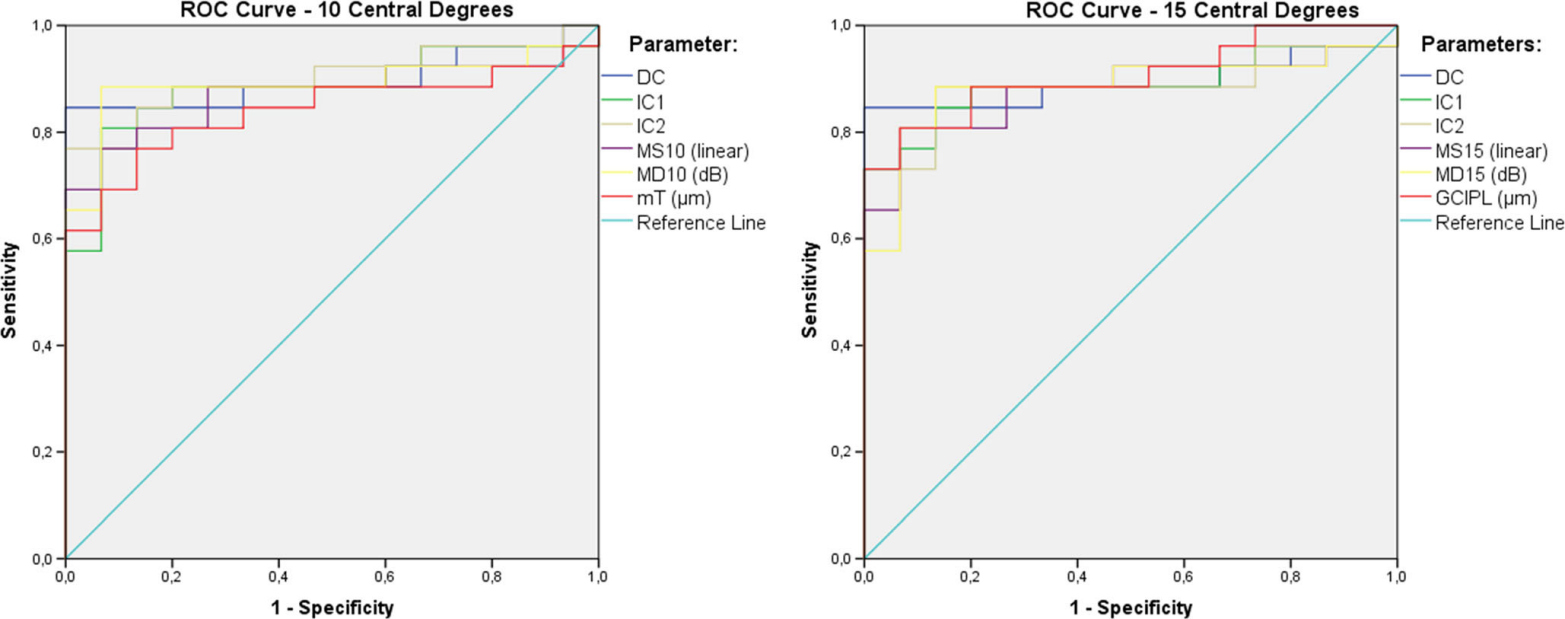
Graphic representation from ROC curve analysis from all parameters evaluated in this study. Left: averages from the 10 central degrees; right: averages from the 15 central degrees. Legend: DC: direct component; IC1: first induced component; IC2: second induced component; MS: mean sensitivity in linear units; MD: mean defect in dB; mT: total macula thickness; GCIPL: ganglion cell-inner plexiform layer thickness; *10° and *15°: averaged from the central 10 and 15 degrees, respectively

In the ROC analysis (Fig. 4), the mfERG RMS10° components (DC and IC2) together with GCIPL presented the highest AUC values (0.897, 0.903, and 0.905 respectively, Table 4). DeLong test did not show any statistical difference between the different AUC shown in Table 4, demonstrating comparable sensitivity and specificity of these structural and functional tests. In the individual analysis, the central 2F–mfERG appeared to show changes prior to those occurring in the central visual field. Linear mixed effects analysis showed a significant association between the RMS10° and mT, but not GCIPL and RMS15°, independent of the presence of a central field defect (MD10°, MD15°).

**Table 4:** Area under the age-adjusted ROC curves (AUC) values from each diagnostic method in the 10° and 15° central area: 2F–mfERG: DC (direct component), IC1, and IC2 (first and second induced components) from the RMS response average of the mfERG; MS and MD: mean linear sensitivity and mean defect calculated considering displacement of the retinal ganglion cells in relation to the function test points. OCT: mT (total macula thickness) and GCIPL (ganglion cell-plexiform layer thickness)

Structure/Function Parameter		AUC (Std. Error)
10 central degrees		
2F–mfERG (RMS 10°)	DC	0.897 (0.052)
	IC1	0.885 (0.054)
	IC2	0.903 (0.049)
MS10° (linear)		0.872 (0.058)
MD10°		0.890 (0.055)
mT		0.841 (0.063)
15 central degrees		
2F–mfERG (RMS 15°)	DC	0.892 (0.055)
	IC1	0.887(0.054)
	IC2	0.874 (0.057)
MS15°(linear)		0.879 (0.055)
MD15°		0.885 (0.056)
GCIPL		0.905 (0.046)

## Discussion

In this study, we assessed the structure function association between OCT, standard automated perimetry, and 2F–mfERG in glaucoma patients and controls with an emphasis on the central 15°. The observed average MD in POAG (4.7, range 2.7/6.7 dB) and in PPG (−0.08, range − 0.6/0.4 dB) reflects our intention to evaluate the function structure association in early glaucoma. In the central retinal response averages both, GCIPL and 2F–mfERG taken alone could separate early PPG as well as POAG patients from control with a comparable performance, but mT and SAP could not.

Our findings complement previous reports of localized macular dysfunction using the mfERG in glaucoma [18, 31, 42]. Macular morphology has also been shown to be affected, with mT demonstrating a lower capability to correctly diagnose glaucoma in comparison with other structure parameters [43, 44].

SAP compared differently to morphologic measures than to functional measures: compared to OCT, SAP had a more significant relationship in regard to the mean defect while when compared to the 2F-mfERG the SAP compared better with respect to mean sensitivity. Previous studies found correlations between the MD [13] or MS [27] of the SAP and mfERG. Others have found no correlation between mean deviation [34] or MS [45] and the mfERG in glaucoma. Those results together with the results of the present study suggest that both MD and MS are important, complementing parameters for structure function analysis. Severity of disease or retinal area analyzed may influence this relationship.

We could confirm previous reports that the 2F–mfERG paradigm can differentiate between glaucoma and controls in the central retina [14, 18, 34, 35]. Including 2F–mfERG and a separate analysis of GCIPL increased the sensitivity to detect retinal abnormalities in individual patients with early glaucoma. Of 26 patients, 30.7% had central mfERG and GCIPL alterations, but no SAP defect in the central 15 degrees. Six patients had a GCIPL thinning with a normal mT measure, four of which had a pathologic mfERG but normal central fields. These findings are in agreement with a previous report that showed that GCIPL thinning in the presence of a normal mT can be detected in areas without glaucomatous field loss [9]. In the central retina, both mfERG and SAP correlated better to mT than to GCIPL. In the 2F–mfERG, mT was associated significantly with DC and IC-2 in all subgroups. GCIPL showed a significant positive correlation to IC2 and a positive trend to DC only in PPG.

Different sensitivities found may be due to individual thresholds. Hood and Kardon argued that when OCT was compared to SAP, the first examination to show alterations would depend on the pre-existing RNFL thickness or SAP sensitivity for each individual patient, prior to the onset of disease [8]. This has been supported by Anraku et al., who demonstrated that patients with a thinner baseline macular ganglion cell complex showed a faster disease progression compared to others [46].

We found that mfERG and GCIPL have the highest predictive diagnostic performance. Thus, our data confirm that a combination of structural and different functional tests increases diagnostic sensitivity in glaucoma. This has also been shown for other patients: In neuro-ophthalmological patients with a field defect, functional retinal changes may be documented with the mfERG, prior to structural changes seen on OCT [47]. Including segmentation of retinal layers, sensitivity of the OCT increased, but still, a fair number of patients had normal appearing retinal layers although retinal dysfunction could be documented with the mfERG [48].

Considering the addition of mfERG in early glaucoma diagnosis we are in agreement with a recent editorial by Medeiros and Tatham, who reviewed results from long term research on structure and function, trying to identify a single diagnostic exam capable of identifying glaucoma first. They conclude that combining different structural and functional measurements outperform the individual test, and that research needs to now focus on how to better integrate these various results to improve diagnosis and follow up in glaucoma [49].

We have shown the mfERG to be very sensitive in early POAG even in patients without a detectable field defect in the central area, and also in PPG, where the SAP is still normal. This adds important information to other reports in which the global flash mfERG identified retinal dysfunction in the periphery (outside the 19 central degrees) of so far unaffected eyes of POAG and ocular hypertension patients [32, 33]. Indeed, electrophysiology can detect early glaucomatous retinal dysfunction while it is still reversible [42].

In our study we were able to recruit six patients with PPG. While this is a small number, these patients still contribute valuable information as they represent the earliest identifiable stage of glaucoma which is diagnosed only by early disc changes.

Most of our control subjects were female while the glaucoma subjects were male. In order to rule out a gender bias, all calculations were adjusted for gender. However, gender differences have been described in total macular thickness, but not in the GCIPL [50], which is the layer where our glaucoma patients differed from control.

Another consideration in our study is the age difference between groups. As there is a known influence of age on the standard mfERG [51–53] and on the GCIPL [50] age and gender were included as “fixed effects” in our mixed–effects models. This balances out the influence of given covariates (here age and gender), which otherwise could be confounded with the target variable.

In our study all examinations were taken on the same day in most eyes (*n* = 19). When this was not possible (*n* = 7) the interval between examinations did not exceed 6 months. In structure-function analysis of glaucoma, measures taken within a half year interval seem generally accepted [54–56]. Also, a follow-up in glaucoma found no significant changes in either SAP or mfERG over this period of time [13]. Thus while progression of disease may influence measures not taken at the same point in time, the slow progression of disease in IOP controlled glaucoma indicates, that the median time interval of 2 months in seven of the patients would only be a minor influence on our results.

### Conclusion

To the best of our knowledge, this is the first study to investigate the association between 2F–mfERG and macular layer segmentation in humans in the central 15°. Adding both the GCIPL analysis and the 2F–mfERG increased the diagnostic performance to detect glaucomatous dysfunction in early glaucoma, including preperimetric glaucoma.

## References

[1] Bourne RR, Stevens GA, White RA, Smith JL, Flaxman SR, Price H, Jonas JB, Keeffe J, Leasher J, Naidoo K, Pesudovs K, Resnikoff S, Taylor HR (2013). Causes of vision loss worldwide, 1990-2010: a systematic analysis. Lancet Glob Health.

[2] Malik R, Swanson WH, Garway-Heath DF (2012). 'Structure-function relationship' in glaucoma: past thinking and current concepts. Clinical & experimental ophthalmology.

[3] Quigley HA, Dunkelberger GR, Green WR (1989). Retinal ganglion cell atrophy correlated with automated perimetry in human eyes with glaucoma. Am J Ophthalmol.

[4] Yoon M, Park S, Kim C, Chin H, Kim N (2014). Glaucoma diagnostic value of the total macular thickness and ganglion cell-inner plexiform layer thickness according to optic disc area. Br J Ophthalmol.

[5] Medeiros FA, Zangwill LM, Alencar LM, Bowd C, Sample PA, Susanna R, Weinreb RN (2009). Detection of glaucoma progression with stratus OCT retinal nerve fiber layer, optic nerve head, and macular thickness measurements. Invest Ophthalmol Vis Sci.

[6] Mwanza JC, Durbin MK, Budenz DL, Sayyad FE, Chang RT, Neelakantan A, Godfrey DG, Carter R, Crandall AS (2012). Glaucoma diagnostic accuracy of ganglion cell-inner plexiform layer thickness: comparison with nerve fiber layer and optic nerve head. Ophthalmology.

[7] Sung MS, Yoon JH, Park SW (2014). Diagnostic validity of macular ganglion cell-inner plexiform layer thickness deviation map algorithm using cirrus HD-OCT in preperimetric and early glaucoma. J Glaucoma.

[8] Hood DC, Anderson SC, Wall M, Kardon RH (2007). Structure versus function in glaucoma: an application of a linear model. Invest Ophthalmol Vis Sci.

[9] Takagi ST, Kita Y, Yagi F, Tomita G (2012). Macular retinal ganglion cell complex damage in the apparently normal visual field of glaucomatous eyes with hemifield defects. J Glaucoma.

[10] Hood DC, Kardon RH (2007). A framework for comparing structural and functional measures of glaucomatous damage. Prog Retin Eye Res.

[11] Pang JJ, Frankfort BJ, Gross RL, Wu SM (2015). Elevated intraocular pressure decreases response sensitivity of inner retinal neurons in experimental glaucoma mice. Proc Natl Acad Sci U S A.

[12] Palmowski AM, Allgayer R, Heinemann-Vernaleken B, Ruprecht KW (2002). Multifocal electroretinogram with a multiflash stimulation technique in open-angle glaucoma. Ophthalmic Res.

[13] Palmowski AM, Ruprecht KW (2004). Follow up in open angle glaucoma. A comparison of static perimetry and the fast stimulation mfERG. Multifocal ERG follow up in open angle glaucoma. Documenta ophthalmologica Advances in ophthalmology.

[14] Palmowski-Wolfe AM, Todorova MG, Orguel S, Flammer J, Brigell M (2007). The 'two global flash' mfERG in high and normal tension primary open-angle glaucoma. Documenta ophthalmologica Advances in ophthalmology.

[15] Ledolter AA, Kramer SA, Todorova MG, Schotzau A, Palmowski-Wolfe AM (2013). The effect of filtering on the two-global-flash mfERG: identifying the optimal range of frequency for detecting glaucomatous retinal dysfunction. Documenta ophthalmologica Advances in ophthalmology.

[16] Ledolter AA, Monhart M, Schoetzau A, Todorova MG, Palmowski-Wolfe AM (2015). Structural and functional changes in glaucoma: comparing the two-flash multifocal electroretinogram to optical coherence tomography and visual fields. Documenta ophthalmologica Advances in ophthalmology.

[17] Kaneko M, Machida S, Hoshi Y, Kurosaka D (2014) Alterations of Photopic negative response of multifocal Electroretinogram in patients with glaucoma. Curr Eye Res:1–10. doi:10.3109/02713683.2014.915575 10.3109/02713683.2014.915575 24832792

[18] Parisi V, Ziccardi L, Centofanti M, Tanga L, Gallinaro G, Falsini B, Bucci MG (2012). Macular function in eyes with open-angle glaucoma evaluated by multifocal electroretinogram. Invest Ophthalmol Vis Sci.

[19] Sutter eET D (1992). The field topography of ERG components in man - I the Photopic luminance response. Vis Res.

[20] Sutter EE, Bearse MA (1999). The optic nerve head component of the human ERG. Vis Res.

[21] Luo X, Patel NB, Harwerth RS, Frishman LJ (2011). Loss of the low-frequency component of the global-flash multifocal electroretinogram in primate eyes with experimental glaucoma. Invest Ophthalmol Vis Sci.

[22] Shimada Y, Bearse MA, Sutter EE (2005). Multifocal electroretinograms combined with periodic flashes: direct responses and induced components. Graefe’s archive for clinical and experimental ophthalmology = Albrecht von Graefes Archiv fur klinische und experimentelle Ophthalmologie.

[23] Hood DC, Frishman LJ, Saszik S, Viswanathan S (2002). Retinal origins of the primate multifocal ERG: implications for the human response. Invest Ophthalmol Vis Sci.

[24] Hood DC, Greenstein V, Frishman L, Holopigian K, Viswanathan S, Seiple W, Ahmed J, Robson JG (1999). Identifying inner retinal contributions to the human multifocal ERG. Vis Res.

[25] Hood DC, Frishman LJ, Viswanathan S, Robson JG, Ahmed J (1999). Evidence for a ganglion cell contribution to the primate electroretinogram (ERG): effects of TTX on the multifocal ERG in macaque. Vis Neurosci.

[26] Luo X, Patel NB, Rajagopalan LP, Harwerth RS, Frishman LJ (2014). Relation between macular retinal ganglion cell/inner plexiform layer thickness and multifocal electroretinogram measures in experimental glaucoma. Invest Ophthalmol Vis Sci.

[27] Kato F, Miura G, Shirato S, Sato E, Yamamoto S (2015). Correlation between N2 amplitude of multifocal ERGs and retinal sensitivity and retinal nerve fiber layer thickness in glaucomatous eyes. Doc Ophthalmol.

[28] Fortune B, Wang L, Bui BV, Cull G, Dong J, Cioffi GA (2003). Local ganglion cell contributions to the macaque electroretinogram revealed by experimental nerve fiber layer bundle defect. Invest Ophthalmol Vis Sci.

[29] Palmowski AM, Allgayer R, Heinemann-Vemaleken B (2000). The multifocal ERG in open angle glaucoma--a comparison of high and low contrast recordings in high- and low-tension open angle glaucoma. Documenta ophthalmologica advances in ophthalmology.

[30] Shimada Y, Li Y, Bearse MA, Sutter EE, Fung W (2001). Assessment of early retinal changes in diabetes using a new multifocal ERG protocol. Br J Ophthalmol.

[31] Miguel-Jimenez JM, Boquete L, Ortega S, Rodriguez-Ascariz JM, Blanco R (2010). Glaucoma detection by wavelet-based analysis of the global flash multifocal electroretinogram. Med Eng Phys.

[32] Chu PH, Chan HH, Brown B (2007). Luminance-modulated adaptation of global flash mfERG: fellow eye losses in asymmetric glaucoma. Invest Ophthalmol Vis Sci.

[33] Chu PH, Ng YF, To CH, So KF, Brown B, Chan HH (2012). Luminance-modulated adaptation in the global flash mfERG: a preliminary study of early retinal functional changes in high-risk glaucoma patients. Graefe’s archive for clinical and experimental ophthalmology = Albrecht von Graefes Archiv fur klinische und experimentelle Ophthalmologie.

[34] Chu PH, Chan HH, Brown B (2006). Glaucoma detection is facilitated by luminance modulation of the global flash multifocal electroretinogram. Invest Ophthalmol Vis Sci.

[35] Kramer SA, Ledolter AA, Todorova MG, Schotzau A, Orgul S, Palmowski-Wolfe AM (2013). The 2-global flash mfERG in glaucoma: attempting to increase sensitivity by reducing the focal flash luminance and changing filter settings. Documenta ophthalmologica Advances in ophthalmology.

[36] Drasdo N, Millican CL, Katholi CR, Curcio CA (2007). The length of Henle fibers in the human retina and a model of ganglion receptive field density in the visual field. Vis Res.

[37] Hood DC, Raza AS (2011). Method for comparing visual field defects to local RNFL and RGC damage seen on frequency domain OCT in patients with glaucoma. Biomedical optics express.

[38] Hood DC, Bach M, Brigell M, Keating D, Kondo M, Lyons JS, Marmor MF, McCulloch DL, Palmowski-Wolfe AM, International Society For Clinical Electrophysiology of V (2012). ISCEV standard for clinical multifocal electroretinography (mfERG) (2011 edition). Documenta ophthalmologica Advances in ophthalmology.

[39] Curcio CA, Allen KA (1990). Topography of ganglion cells in human retina. J Comp Neurol.

[40] R: A Language and Environment for Statistical Computing (2016) 3.0.2 edn. R Foundation for Statistical Computing, Vienna, Austria

[41] Hanley JA, McNeil BJ (1982). The meaning and use of the area under a receiver operating characteristic (ROC) curve. Radiology.

[42] Wilsey LJ, Fortune B (2016). Electroretinography in glaucoma diagnosis. Curr Opin Ophthalmol.

[43] Medeiros FA, Zangwill LM, Bowd C, Vessani RM, Susanna R, Weinreb RN (2005). Evaluation of retinal nerve fiber layer, optic nerve head, and macular thickness measurements for glaucoma detection using optical coherence tomography. Am J Ophthalmol.

[44] Wollstein G, Ishikawa H, Wang J, Beaton SA, Schuman JS (2005). Comparison of three optical coherence tomography scanning areas for detection of glaucomatous damage. Am J Ophthalmol.

[45] Fortune B, Bearse MA, Cioffi GA, Johnson CA (2002). Selective loss of an oscillatory component from temporal retinal multifocal ERG responses in glaucoma. Invest Ophthalmol Vis Sci.

[46] Anraku A, Enomoto N, Takeyama A, Ito H, Tomita G (2014). Baseline thickness of macular ganglion cell complex predicts progression of visual field loss. Graefe’s archive for clinical and experimental ophthalmology = Albrecht von Graefes Archiv fur klinische und experimentelle Ophthalmologie.

[47] Dale EA, Hood DC, Greenstein VC, Odel JG (2010). A comparison of multifocal ERG and frequency domain OCT changes in patients with abnormalities of the retina. Documenta ophthalmologica Advances in ophthalmology.

[48] Talamini CL, Raza AS, Dale EA, Greenstein VC, Odel JG, Hood DC (2011). Abnormal multifocal ERG findings in patients with normal-appearing retinal anatomy. Documenta ophthalmologica Advances in ophthalmology.

[49] Medeiros FA, Tatham AJ (2016). Structure versus function in glaucoma: the debate that Doesn’t need to be. Ophthalmology.

[50] Ooto S, Hangai M, Yoshimura N (2015). Effects of sex and age on the normal retinal and choroidal structures on optical coherence tomography. Curr Eye Res.

[51] Mohidin N, Yap MK, Jacobs RJ (1999). Influence of age on the multifocal electroretinography. Ophthalmic & physiological optics : the journal of the British College of Ophthalmic Opticians (Optometrists).

[52] Palmowski-Wolfe AM, Woerdehoff U (2005). A comparison of the fast stimulation multifocal-ERG in patients with an IOL and control groups of different age. Documenta ophthalmologica Advances in ophthalmology.

[53] Nabeshima T, Tazawa Y, Mita M, Sano M (2002). Effects of aging on the first and second-order kernels of multifocal electroretinogram. Jpn J Ophthalmol.

[54] Raza AS, Cho J, de Moraes CG, Wang M, Zhang X, Kardon RH, Liebmann JM, Ritch R, Hood DC (2011). Retinal ganglion cell layer thickness and local visual field sensitivity in glaucoma. Arch Ophthalmol.

[55] Hori N, Komori S, Yamada H, Sawada A, Nomura Y, Mochizuki K, Yamamoto T (2012). Assessment of macular function of glaucomatous eyes by multifocal electroretinograms. Documenta ophthalmologica Advances in ophthalmology.

[56] Hood DC, Harizman N, Kanadani FN, Grippo TM, Baharestani S, Greenstein VC, Liebmann JM, Ritch R (2007). Retinal nerve fibre thickness measured with optical coherence tomography accurately detects confirmed glaucomatous damage. Br J Ophthalmol.

